# Editorial: The Potential Effect and Mechanism of Chinese Traditional Medicine on Vascular Homeostasis and Remodeling

**DOI:** 10.3389/fphar.2020.599766

**Published:** 2020-12-17

**Authors:** Yuliang Wang, Yuefan Zhang, Tie-Jun Li, Ismail Laher, Hongbin Wang

**Affiliations:** ^1^ Joint International Research Laboratory of Metabolic and Developmental Sciences, Key Laboratory of Urban Agriculture (South) Ministry of Agriculture, Plant Biotechnology Research Center, Fudan-SJTU-Nottingham Plant Biotechnology R&D Center, School of Agriculture and Biology, Shanghai Jiao Tong University, Shanghai, China; ^2^ Biomedical Innovation R&D Center, School of Medicine, Shanghai University, Shanghai, China; ^3^ Department of Pharmacology, School of Pharmacy, Navy Medical University, Shanghai, China; ^4^ Department of Pharmacology and Therapeutics, Faculty of Medicine, University of British Columbia, Vancouver, BC, Canada; ^5^ Center for Biomedical Informatics, Texas A&M University Health Science University, Houston, TX, United States

**Keywords:** Chinese traditional medicine, vascular homeostasis and remodeling, clinical effect, mechanism, natural product

Vascular diseases, such as coronary artery disease and stroke, are the leading cause of CVD deaths (Thomas et al., 2018) and mortality related to vascular diseases will continue to increase over the next 10 years (Liu et al., 2019). Cardiovascular diseases result from alterations in the homeostatic balance of vascular structure and function. Traditional Chinese Medicine (TCM) has long been proposed as an effective treatment for cardiovascular disease. Many formulae, herbs and natural products, such as Long-Dan-Xie-Gan Tang (formula), Danshen (herb) Bao et al., Huanglansu (natural product), have been investigated for their effects in the prevention and treatment of cardiovascular diseases by regulating the vascular homeostasis and remodeling, for example by scavenging free radicals, inhibiting inflammatory reaction, reducing oxidative stress and modulating apoptosis.

A study by Zhang et al. reviewed 27 high-quality randomized controlled trials (RCT) that evaluated the efficacy and safety of Chinese herbal medicines for coronary heart disease (CHD). The cardio-protection of TCM are likely related to its ability to inhibit inflammation, oxidative stress, apoptosis, improve circulatory function, and stimulate energy metabolism (Zhang et al.).

Ischemic stroke is another major cause of death and disability globally. The predominant risk factor in ischemic stroke is mitochondrial dysfunction caused by an imbalance of mitochondrial fusion and fission. A study by Gao et al. reports that C-phycocyanin (C-pc), an active component of *Spirulina platensis*, can affect mitochondrial fission and fusion dynamics and reduce apoptosis in cardiomyocytes, suggesting that C-pc may be used to protect the ischemic heart disease (Gao et al.).

Hypoxia-inducible factor-1 (HIF-1) has been suggested as a target when treating ischemic stroke. Levels of HIF-1 are increased under hypoxic conditions and regulate the adaptive responses to hypoxia-induced angiogenesis. Several preclinical studies report that TCM that target HIF-1 can be used either as anti-angiogenic agents (for example, to treat tumors) or as pro-angiogenic agents (for example, to treat stroke). The dual effects of TCM on the HIF-1 pathway make it suitable for the treatment of ischemic stroke and cancer (Hong et al.).

Danshensu is a water-soluble phenolic acid found in *Salvia miltiorrhiza*. Several studies reported that danshensu has unique bioactivity on acute myocardial ischemia injury. A report by Meng et al. described the pharmacokinetic profile (absorption, distribution, metabolism, and elimination profiles) of sodium danshensu in rats. Danshensu is poorly absorbed, widely distributed, bio-transformed via several metabolic pathways, and is excreted mainly in the urine (Meng et al.).

The majority (80%) of strokes are stroke are due to focal brain ischemia. Restoring blood flow is necessary to limit irreversible damage to brain tissue, but events related to cerebral ischemia reperfusion (I/R) can lead to an insufficient oxygen supply and restoration of blood flow, where the imbalance of brain energy levels causes further neuroinflammation, neuronal damage, and cerebral edema. A study by Li et al. demonstrates that stachydrine down-regulates inflammation, restores neurological function, reduces neuronal injury and cerebral infarction in a rat model of ischemic stroke. The antioxidant and anti-inflammatory effects of stachydrine could prevents reperfusion-induced injury in patients with stroke (Li et al.). Another study by Wang et al. indicates that kaempferol can protect against I/R induced brain damage *in vivo.* Kaempferol increases the activity of antioxidant pathways related to SOD and GSH, while also decreasing serum and brain tissue content of MDA by regulating the expression of proteins related to oxidative and inflammatory stress (Wang et al.).

Blood vessels are composed of multilayered cells with unique histological, biochemical, and functional characteristics. Each layer maintains vascular homeostasis and regulates vascular remodeling caused by hemodynamic stress or vascular injury. Alterations in vascular homeostasis are associated with a number of deleterious changes in the cardiovascular system, and many TCM can maintain vascular stability and prevent vascular dysfunction.

Bird’s nest is formed by the saliva of swiftlets, and is used as both a medicine and as food in China. The aqueous extract of hydrolyzed edible bird nest (HBN) was examined on diabetic endothelial dysfunction *in vitro* and *in vivo*. HBN is able to protect against high-glucose induced endothelial dysfunction by restraining oxidative stress and increasing NO bioavailability (Murugan et al.). Caffeic acid phenethyl ester inhibits activation of the AKT/NF-κB pathway and NLRP3 inflammasome, and is a potential natural product for the prevention of calcific aortic valve disease (Liu et al.).

Traditional Chinese medicines can also be also used to treat vascular diseases other than CHD and stroke. Patients with CHD and blood stasis syndrome often suffer from angina pectoris which is sometimes difficult to treat due to the lack of effective drugs. A study by Ma et al. used deoxynojirimycin, a unique polyhydroxy alkaloid found in mulberry leaves, to treat patients with stable angina pectoris in a clinic trial involving 144 patients. Deoxynojirimycin improved the clinical symptoms in patients with stable angina pectoris by increasing antioxidant and anti-inflammatory capacities (Ma et al.). Anisodamine, a belladonna alkaloid, decreased serum potassium and on-site mortality in crush syndrome through activation of α7nAChR (Yu et al.).

The history and use of TCM go back thousands of years, and it is clear that TCM is a huge treasure trove awaiting research of new drug discovery. This *Research Topic* focuses on the effects and mechanisms of TCM in different vascular diseases, based on data from human and animal studies, and in isolated cells. These studies allowed for a description of the molecular mechanisms of action of many active compounds of TCM.

Traditional Chinese medicine is unique in its therapeutic approach in that it is often used as a combination with other herbs to treat a range of diseases, although it is still difficult to clarify the mechanisms of action of traditional TCM. The multi-target effects of TCM provides many therapeutic benefits and understanding the scale and nature of these effects will derive from a systems biology approach coupled with modern molecular techniques including proteomics (Li and Su, 2008; Gu and Pei 2017; Cai et al., 2018). A large scale evidence based approach using TCM candidates with therapeutic effects could reduce the global epidemic of cardiovascular health challenges.

## Author Contributions

Correspondence: YW, wangyuliang@sjtu.edu.cn YW wrote the article, IL edited the article and YZ revised the article.

## Conflict of Interest

The authors declare that the research was conducted in the absence of any commercial or financial relationships that could be construed as a potential conflict of interest.
